# Spinal Cord Injury From an Epidural Abscess as a Serious Complication of COVID-19 Infection

**DOI:** 10.7759/cureus.11327

**Published:** 2020-11-04

**Authors:** Peter Soh, Ninh Doan, Bailey Manning, Hayley Doan

**Affiliations:** 1 Neurology, University of South Alabama, Mobile, USA; 2 Neurosurgery, Baptist South Hospital, Montgomery, USA

**Keywords:** spinal cord injury, covid-19, covid-19 neurological outcomes, epidural abscess

## Abstract

A 53-year-old female admitted to the hospital for generalized weakness, fever, and cough, tested positive for coronavirus disease 2019 (COVID-19). She experienced cardiac arrest and then developed a deep-venous thrombosis and pneumonia. She then developed new-onset paraplegia due to an epidural abscess found on thoracic-spine imaging. After surgical removal of the epidural abscess, the patient improved clinically. This is a unique case report of a patient developing paraplegia secondary to an epidural abscess as a serious complication of COVID-19 infection.

## Introduction

As of July 2020, there have been 901 recorded cases of coronavirus disease 2019 (COVID-19) with neurological symptoms possibly due to a combination of neuro-invasive properties of the virus and downstream effects from multi-organ dysfunction and altered biochemistry [1,2]. Common neurological symptoms included olfactory and gustatory disorders. Guillain-Barré syndrome and acute inflammation of the brain, spinal cord, and meninges were reported.

## Case presentation

**Figure 1:**
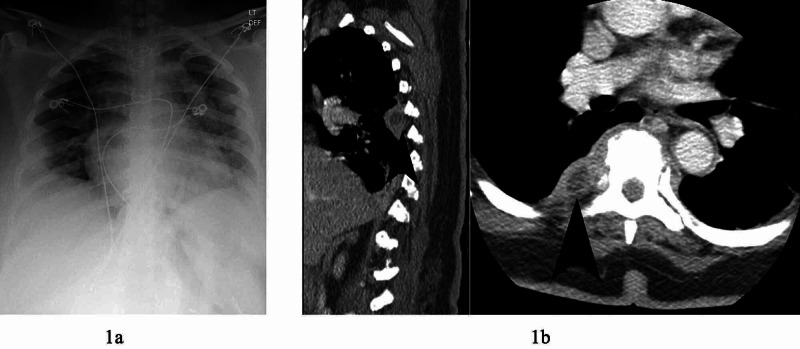
Chest x-ray and CT chest with contrast. 1a: Plain chest x-ray showing decreased lung volume, cardiomegaly, and multiple opacities concerning of coronavirus disease 2019 (COVID-19) infection. 1b: CT chest with contrast demonstrating ring enhancing lesions (arrows) at base of right lung at thoracic vertebrae 5 and 6 in sagittal and axial views.

We present a 53-year-old female, with hypertension and obesity, who was admitted to the hospital for generalized weakness, fever, and cough in May 2020. On admission, her laboratory results showed: white blood cells (WBCs) 3.7 x 10-3/ul (reference: 4.1-10.3 x 10-3/ul), platelets 213 x 10-3/ul (reference: 140-400 x 10-3), and international normalized ratio (INR) 1.03. Chest x-ray showed pneumonia (Figure 1a). Nasopharyngeal sample tested positive for COVID-19 using reverse transcription polymerase chain reaction (RT-PCR). She required supplementary oxygen and then by day four required intubation and mechanical ventilation for respiratory distress. 

Due to her medical deterioration, the patient was treated with plasma therapy. She improved and was extubated on day 18, but subsequently struggled to breathe and went into asystole requiring cardiopulmonary resuscitation and re-intubation. Ultrasound showed right lower-extremity deep vein thrombosis (DVT) on day 24. The patient was treated with full-dose lovenox for anticoagulation. 

On day 25, her sputum and blood cultures were positive for *Serratia marcescens*, and she was treated with meropenem for 14 days for *Serratia* bacteremia pneumonia. The next day, D-dimer was elevated to 12, and patient was transitioned to apixaban. On day 30, she was extubated, and six days later, the patient developed paraplegia. Prior to admission, she denied neurological deficits or back pain. CT of lumbar and thoracic spine showed a 1.6 cm ring-enhancing collection in the right lung concerning for abscess at the T5-T6 vertebral levels (Figure 1b). 

**Figure 2:**
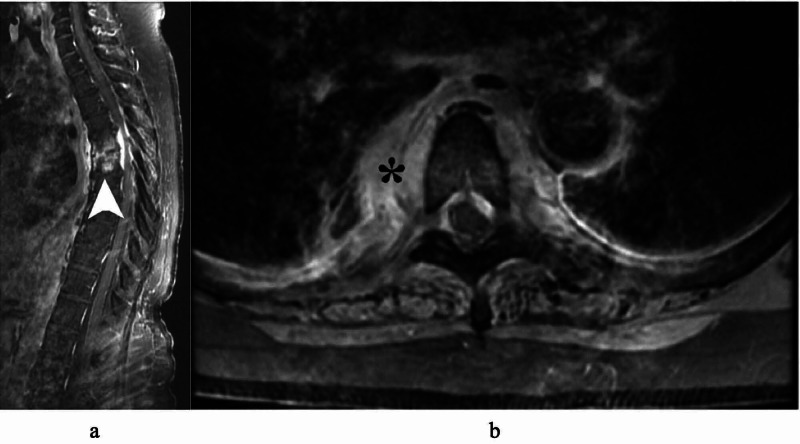
MRI thoracic spine T1 with contrast in sagittal (a) and axial (b) displaying T5-6 body and epidural enhancements (arrow) concerning for osteodiscitis and epidural abscess. There is paraspinal enhancement (asterisk) adjacent to the lung.

MRI of thoracic spine with contrast showed circumferential enhancement around the T5 and T6 vertebral bodies, and moderate anterior-posterior epidural enhancement with cord-compression consistent with epidural abscess (Figure 2). 

She was taken for washout, fusion, and decompression of the affected spine by neurosurgery. Purulent discharge and phlegmon with significant stenosis were noted at T5-6 epidural space. Samples from surgery grew *Staphylococcus capitis*. Post-operatively, she had improvement in lower extremity strength and sensation. She was transferred to a rehabilitation facility, and at one-month follow-up she was able to move her lower extremities. 

## Discussion

As of October 21, 2020, there have been a total of 40,886,174 cases confirmed and 1,126,379 COVID-19 deaths worldwide [3]. In a case series of 214 patients with COVID-19 from Wuhan, China, 36% had neurologic symptoms [4]. Coagulopathy also is a major pathological occurrence in COVID-19 patients, and markers of coagulopathy, such as elevated D-dimer, may indicate a poor prognosis [5]. One retrospective analysis found that anticoagulant therapy, primarily with low molecular weight heparin, was associated with lower 28-day mortality in patients with abnormal coagulation parameters; elevated D-dimer level or sepsis-induced coagulopathy score [6]. 

Another case series of 184 ICU patients with COVID-19 pneumonia demonstrated a 31% incidence of thrombotic complications, including deep vein thrombosis, pulmonary embolism, and ischemic stroke. The study suggested the importance of low-dose thrombosis prophylaxis in all COVID-19 patients admitted to the ICU [7]. In another retrospective analysis, 83% of COVID-19 patients also had lymphocytopenia at hospital admission [8]. 

Ventilator-associated pneumonia (VAP) is the second most common nosocomial infection [9]. In one analysis evaluating bacterial pathogens of VAP, *Serratia marcescens* was identified in 2% of patients, while *Staphylococcus aureus* was the most common, found in 44% of patient cultures [10]. *Serratia* is not a common source of pneumonia (4.1% incidence in the U.S.) and can be associated with iatrogenic infection [11,12]. 

## Conclusions

In this case report, the patient was lymphocytopenic and coagulopathic in the setting of COVID-19 infection. She developed pneumonia from COVID-19 that spread to the nearby spine resulting in the spinal epidural abscess. Figure 1b and Figure 2a demonstrate the right lung base abscess in direct connection with the T5-6 spinal epidural abscess compressing the spinal cord. To our knowledge, this is the first case reported of a patient who developed paraplegia secondary to epidural abscess as a serious complication of COVID-19 infection. This case highlights the potential for a myriad of serious complications from this relatively unknown virus due to its ability to elicit a wide range of inflammatory reactions. Close neurological monitoring, including turning off sedation for a more accurate neurological examination, is highly recommended as early diagnosis and treatment can lead to a significantly improved outcome. 
